# iTextMine: integrated text-mining system for large-scale knowledge extraction from the literature

**DOI:** 10.1093/database/bay128

**Published:** 2018-12-14

**Authors:** Jia Ren, Gang Li, Karen Ross, Cecilia Arighi, Peter McGarvey, Shruti Rao, Julie Cowart, Subha Madhavan, K Vijay-Shanker, Cathy H Wu

**Affiliations:** 1 Center for Bioinformatics and Computational Biology, University of Delaware, Newark, DE, USA; 2 Department of Computer and Information Sciences, University of Delaware, Newark, DE, USA; 3 Protein Information Resource, Georgetown University Medical Center, Washington, DC, USA; 4 Innovation Center For Biomedical Informatics, Georgetown University, Washington, DC, USA; 5 Lombardi Comprehensive Cancer Center, Georgetown University Medical Center, Washington, DC, USA

## Abstract

Numerous efforts have been made for developing text-mining tools to extract information from biomedical text automatically. They have assisted in many biological tasks, such as database curation and hypothesis generation. Text-mining tools are usually different from each other in terms of programming language, system dependency and input/output format. There are few previous works that concern the integration of different text-mining tools and their results from large-scale text processing. In this paper, we describe the iTextMine system with an automated workflow to run multiple text-mining tools on large-scale text for knowledge extraction. We employ parallel processing with dockerized text-mining tools with a standardized JSON output format and implement a text alignment algorithm to solve the text discrepancy for result integration. iTextMine presently integrates four relation extraction tools, which have been used to process all the Medline abstracts and PMC open access full-length articles. The website allows users to browse the text evidence and view integrated results for knowledge discovery through a network view. We demonstrate the utilities of iTextMine with two use cases involving the gene PTEN and breast cancer and the gene SATB1.

## Introduction

With the rapid growth of the biomedical literature, text-mining tools have attracted more research interest as they can extract structural information from text automatically. To date, most text-mining tools are specialized to specific tasks and are used to recognize certain types of entities or relations (1–4). It is likely that users may need to combine results from different text-mining tools to extract more comprehensive and/or higher-level knowledge. For example, in iPTMnet (5), three different text-mining tools (2, 3, 6) were used to extract and post-process information for protein post-translational modification. To gather information extracted by different text-mining tools, users often need to run each tool independently as the tools may use different programming languages, formats and system dependencies. It is time-consuming for users to handle these technical details every time when using a particular tool. To address this issue, we developed the iTextMine system with an automated workflow to run multiple text-mining tools on text at large scale for knowledge extraction. The system defines a standardized format for result integration and visualization with a network view. iTextMine currently consists of four in-house developed text-mining tools: (i) RLIMS-P (3) for mining protein phosphorylation (kinase substrate site), (ii) eFIP (6) for phosphorylation-dependent protein–protein interaction (PPI), (iii) miRTex (4) for miRNA–gene relation and (iv) eGARD (7) for gene/protein variant therapeutic response in cancer information from the scientific literature. For gene and other entity normalization, we incorporated results from PubTator (8).

A few natural language processing (NLP) frameworks have been created to improve interoperability and encourage sharing and reusing NLP components (e.g. tokenizer, sentence splitter, Part-Of-Speech (POS) tagger and syntactic parser). Developers arrange different components to support tasks such as named entity recognition (NER) and relation extraction, and they often have a built-in analysis module to evaluate the performance. Here we list some popular systems. General Architecture for Text Engineering (9) is an NLP toolkit including NLP components, visualizing text and perform text evaluation. Alvis (10) is a pipeline framework to annotate text documents using NLP tools. DKPro Core (11) wraps existing NLP tools into Unstructured Information Management Architecture (UIMA) (12) components. Argo (13) is a generic text-mining workbench that integrates NLP/text-mining elementary components. It can be used as a curation platform, and it has greatly facilitated the curation of disease. BeCalm (14), the BiomEdiCAL annotation Metaserver, is a community effort integrating, visualizing and evaluating biomedical entity recognition tools.

Most of the systems are implemented in JAVA, and they define their own extensible markup language (XML) for internal data exchange, such as ATLAS interchange format, UIMA’s common analysis structure and BeCalm’s XML-RPC format. In 2013, the BioC format (15) was proposed to combine these efforts for more powerful and capable sharing of text and annotations. The BioC pipeline (16) showcases that the format is interoperable with existing well-known NLP toolsets.

Unlike these existing NLP frameworks, which are aimed at creating shareable and reusable NLP components and building customized text-mining pipelines from scratch, the iTextMine system is designed to integrate existing full-fledged text-mining tools in different languages with different dependencies and to run the tools on text at large scale. We can apply all the integrated text-mining tools to process the entire set of Medline abstracts and PMC open access full-length articles. The system solves the text discrepancies issues caused by some text-mining systems, so results from different tools can be combined together for better utilization. SemRep (17), BioContext (18) and Turku Event Extraction System (19) are some of the text-mining systems that have been applied over the whole Medline. They can be incorporated into iTextMine as individual tools. iTextMine also provides a user-friendly interface to search and browse a wide range of bio-entities and relations.

We faced three main challenges while integrating different tools for large-scale processing and knowledge integration in iTextMine: (i) text-mining tools are implemented in different languages and have different run-time dependencies. It is cumbersome to maintain these dependencies in the parallel execution engine. Meanwhile, we need to make sure each tool can be run in parallel, e.g. two parallel processes should not write to the same file to avoid conflicts; (ii) each tool has its own output format to describe the extracted information, and it is hard to store these results with the same database schema; and (iii) some text-mining tools modify the original text, and the text offset of entities and relations cannot be matched to the original text, making it impossible to directly compare and combine the results from different tools.

In the rest of the paper, first we describe the methods used to address these three challenges for text-mining tool integration. We then present the results for the large-scale processing using the four relation extraction tools and demonstrate the usage of the iTextMine website for querying the database and for result visualization. Lastly, we show the utility of iTextMine with two use cases: PTEN and breast cancer and regulation and disease involvement of SATB1, a protein involved in regulating chromatin remodeling.

## iTextMine system

### System overview

**Figure 1:**
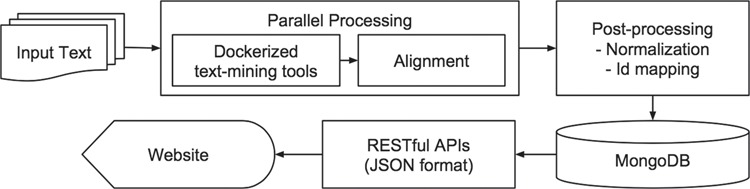
iTextMine system overview.

Figure 1 depicts the iTextMine workflow. First, a parallel execution engine runs text-mining tools on the input text and performs text alignment. The parallel processing is necessary for running the tools on large scale within a smaller amount of time. Text alignment resolves the discrepancies of different text-mining tools by adjusting entity offsets to original input text. Additional post-processing tasks run afterward, such as entity normalization and ID mapping. The post-processed data are imported into a MongoDB. Finally, web services disseminate the results. We created representational state transfer application program interfaces (REST APIs) to serve the data for both front-end web interface and external users. The results are in JSON format and can be converted to other community standard dissemination formats, such as BioC (15) and brat standoff format (20).

**Figure 2:**
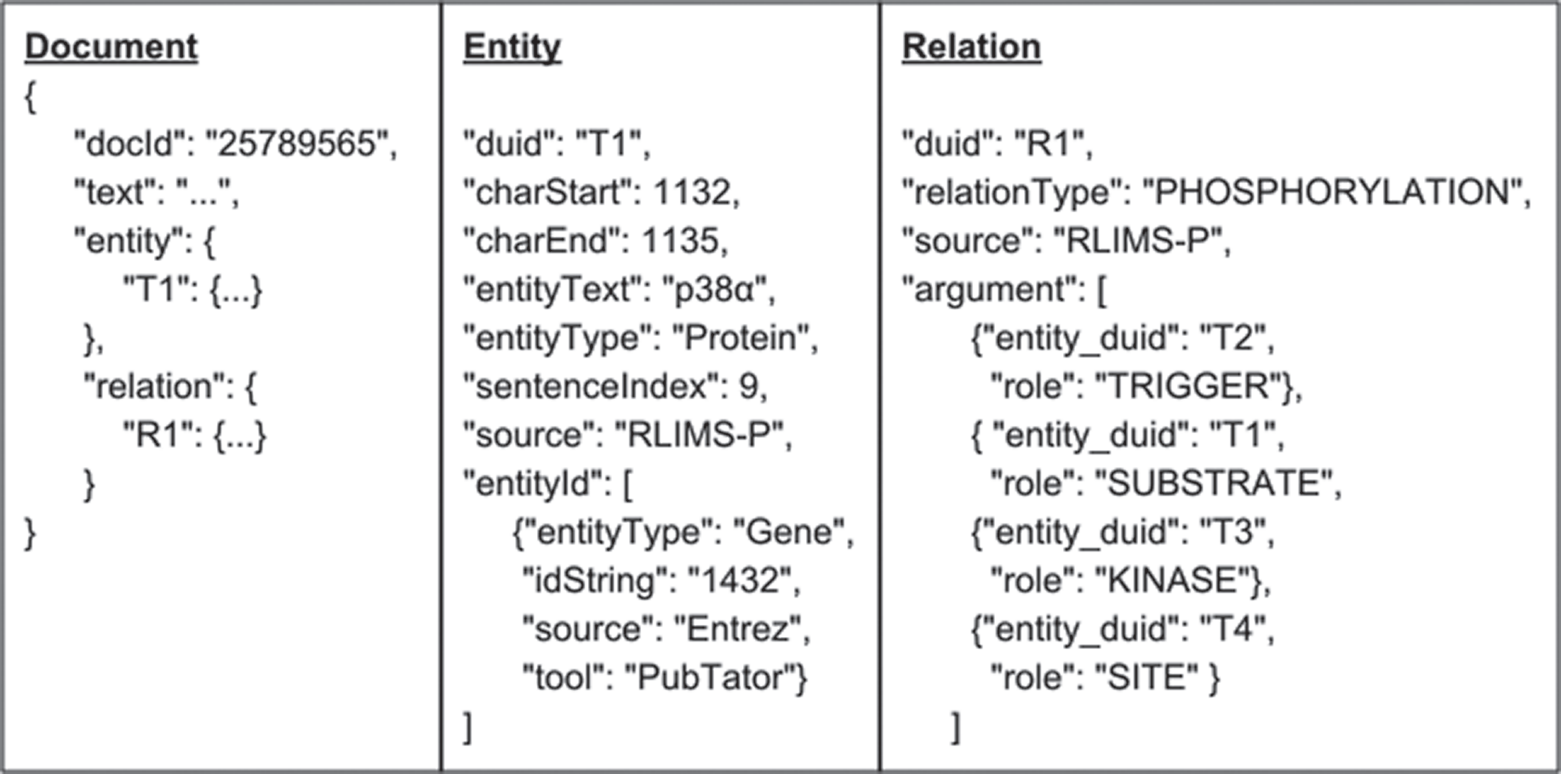
Standardized JSON format.

Next, we will describe each component in detail.

### Parallel processing

In order to process a large number of documents, we run the text-mining tools using a parallel processing engine, Spark (21). We use the PySpark built-in web interface to monitor the processing progress and memory usage. The entire document input collection is broken into small chunks for memory efficiency. The chunk size can be adjusted for each tool. For most of our tools, we process 100 documents per task. The documents are either Medline abstracts or paragraphs from PMC articles.

#### Dockerized text-mining tools

Text-mining applications are containerized with Docker (https://www.docker.com) into individual Linux containers. The performance of a Docker container is nearly identical to its native form (22). The containers can be run with a consistent command and without considering specific programming language and system dependencies. They are also scalable, which means multiple containers are independent of each other and won’t conflict when multiple containers are run at the same time.

#### Text alignment

In the text-mining results, bio-entities are labeled by their offsets in the text. When merging multiple text-mining results, it is often desirable to use offsets to recognize when different text-mining entities refer to the same object. However, during text-mining processing, the original text might be modified by some NLP steps that lead to shifted entity offsets. For example, tokenization might insert an extra space between a word and a punctuation mark, special tags and symbols might be used to mark the text, Unicode characters might be converted to American Standard Code for Information Interchange (ASCII) words (α to alpha) and so on. It is difficult to ask every developer to maintain the original text offsets during their entire workflow. Thus, we use a text alignment algorithm, the Hirschberg’s algorithm (23), to correct the shifted offsets to the original position. This step is run for each tool after text-mining results are generated. The Hirschberg’s algorithm aims to find the optimal sequence alignment between two strings with dynamic programming. Given two sequences, with lengths n and m, Hirschberg’s algorithm calculates the best alignment between the two sequences in O(nm) time with constant space (O(min{n, m})). With the alignment, we then can map the position in the modified text back to the position in the original text.

### Post-processing

Post-processing steps are optional steps to clean up and add additional information for text-mining results. For our four relation extraction tools, we added entity normalized ID and performed ID mapping for extracted relation arguments. The normalization step compares the argument text offsets with normalized entities from PubTator and assigns a database identifier to the matched argument. After normalization, textual variations and synonyms of an entity are mapped to the same identifier. Relations with the same arguments are connected together to form a network. In the iTextMine system, we incorporate entity normalization provided by PubTator (8). PubTator annotates and normalizes genes/proteins, diseases, species, chemicals and gene mutations. When an entity in a relation is not present in the results of Pubtator, we still keep the entity without a normalization ID.

Usually, we use UniProt AC (24) for protein entities, National Center for Biotechnology Information (NCBI) gene ID (25) for miRNA and disease ontology (26) for diseases. Additional NER and normalization sources can be added to the system to include other types of entities. For genes/proteins, PubTator only provides NCBI gene ID, so a second ID mapping post-processing step is run to find the corresponding UniProt accession using UniProt ID mapping service (https://www.uniprot.org/uploadlists).

### Web interface

The user interface is built with the React (https://reactjs.org) framework. The code is well structured and modularized for better reusability. D3.js (https://d3js.org) is used for data visualization to create an interactive network view for the text-mining results. The usage of the web interface will be presented in the result session.

### Standardized JSON format

We adopt a standardized JSON format for iTextMine system (Figure 2). JSON objects can be parsed by a standard built-in function in most modern programming languages. The format can be directly stored in MongoDB and can be directly consumed by front-end web application.

The standardized format is document-centric, i.e. each document contains a doc id field, a text field, a list of properties, a hash table of entities and a hash table of relations. Each entity element contains information such as entity type, offsets and normalization ID, while each relation element contains relation type, source and its arguments.

### Full-scale processing

After building the iTextMine system, we use it to process all of the Medline abstracts and PMC open access full-length articles with the relation extraction tools.

### Text preparation

First, Medline abstracts and PMC articles in XML format are downloaded from the PubMed website. We then convert the XML files into the standardized JSON format. While parsing the PMC XML files, a full article is separated into paragraphs with a unique ID representing their original order in the article. We also keep the section type as an attribute for the paragraph (e.g. introduction, methods, results or conclusion), which can be used to obtain a subset of the text if we are only interested in processing certain section types (e.g. results and discussions). In addition, the captions of tables and figures are extracted as a single paragraph. Each paragraph is a document for the parallel processing pipeline. The current text-mining tools extract relations from a single sentence or a paragraph, so breaking the full article into paragraphs won’t affect the extraction results.

We use Apache Lucene (http://lucene.apache.org) to index the text for flexible queries. It has two main purposes: first, Lucene supports powerful regular expression searches; second, our own index allows searches at the paragraph level for PMC articles.

### Pre-processing

For each relation extraction task, we use a filtering step to obtain a set of possibly positive documents. This minimizes the number of articles that need to be processed and saves processing time. For example, RLIMS-P is a rule-based system that extracts protein phosphorylation relations. As it depends on the existence of a trigger word (e.g. phosphorylation) for relation extraction, we use a trigger word-based query ‘phospho^*^’ to obtain a subset of articles. Text without the trigger word won’t generate any results, thus are safe to remove. For RLIMS-P, with the filter step we can retrieve ~300 000 PubMed abstracts, a much smaller set compared to the entire Medline set of 30 million abstracts.

**Table 1:** iTextMine full-scale processing results of Medline abstracts

**Text-mining tool**	**# Positive abstracts**		**Relation types/counts**	**Duration**
	Entities/triggers	Entities + relations
RLIMS-P	322 955	202 579	phosphorylation (kinase substrate site): 383 413	10.7 h
eFIP	294 915	23 584	phosphorylation-dependent PPI: 35 368	1.9 h
miRTex	158 127	27 462	miRNA target: 33 636	8.1 h
			miRNA–gene regulation: 46 115	
			gene–miRNA regulation: 8328	
eGARD	629 696	40 225	gene–disease drug response: 82 402	47.9 h

**Table 2:** iTextMine full-scale processing results of PMC open access full-length articles

**Text-mining tool**	**# Positive paragraphs**		**Relation types/counts**	**Duration**
	Entities/triggers	Entities + relations
RLIMS-P	645 080	588 693 (in 112 003 articles)	phosphorylation (kinase substrate site): 510 764	22 h
eFIP	588 693	70 250 (in 29 236 articles)	phosphorylation-dependent PPI: 70 250	3.5 h
miRTex	718 927	84 607 (in 20 110 articles)	miRNA target: 96 155	37 h
			miRNA–gene regulation: 129 820	
			gene–miRNA regulation: 21 427	

Similarly, other tool developers may provide specific queries or criteria to filter the input text. For example, eFIP is built upon RLIMS-P and needs to use RLIMS-P results, so we only process RLIMS-P positive articles using eFIP. miRTex extracts miRNA and gene relations, so we run a miRNA entity recognition tool (4) to ensure that each document has at least one miRNA mention. Finally, eGARD extracts associations between gene/protein variants and therapeutic responses to drug from abstracts. To have such associations mentioned in the text, an abstract needs to describe the genomic anomalies and the outcome of drug treatments. We search PubMed for abstracts that mention genes and narrow down the returned set by looking for words or phrases that indicate therapeutic responses (7).

## Results

We downloaded, parsed and imported 28 889 481 Medline abstracts and 2 142 580 PMC open access full-length articles (broken into 71 049 694 paragraphs) on 23 September 2018. The parallel processing pipeline was set up on a machine with 24 CPUs and 64G RAM. We set the processing core number to 10, collected the tool outputs and tracked the time usage. We observed 7–8× speedup using iTextMine system than sequential processing using one CPU core for the four relation extraction tools.

For Medline abstracts, Table 1 summarizes the statistics for each tool—the number of positive abstracts, the counts of the specific relations types extracted and the processing time. Overall, iTextMine identified 265 071 abstracts with at least one relation extracted by its underlying text-mining tools. For PMC open access articles, the statistics are listed in Table 2. eGARD is under development for PMC article processing and hasn’t been applied on PMC articles yet. All extracted results are stored in the database and can be queried using the web interface.

We also summarize the number of Medline abstracts and PMC articles that contain relations extracted by multiple text-mining tools (Table 3). A total of 89.4% Medline abstracts contain relations from one tool, 10.3% have at least two types of relations, 0.18% have three and only one PMID has relations extracted by all the four text-mining tools (PMID: 19633292). For PMC open access full-length articles, 74.08% articles have relations extracted from one tool, 24.80% have two and 1.12% have three.

### Web interface and use cases

The iTextMine web interface provides many features to help biologists explore the text-mining results. Here we describe the main functionalities of the site and demonstrate its utility with two use cases.

#### Search interface

iTextMine website supports querying by keywords, PMIDs for Medline abstracts and PMCIDs for PMC articles. If a keyword query is used, the query will be sent to PubMed to obtain a list of relevant PMIDs. Then the results from the text-mining results are retrieved from the database.

**Table 3:** Medline abstracts and PMC articles with extracted relations

**Number of tools**	**Medline abstracts**		**PMC open access full-length articles**	
1	236 683	89.29%	94 076	74.08%
2	27 998	10.56%	31 499	24.80%
3	393	0.15%	1425	1.12%
4	1	0.00%	0^a^	0.00%
Total	265 075	100.00%	127 000	100.00%

^a^eGARD hasn’t been applied to PMC articles.

**Figure 3:**
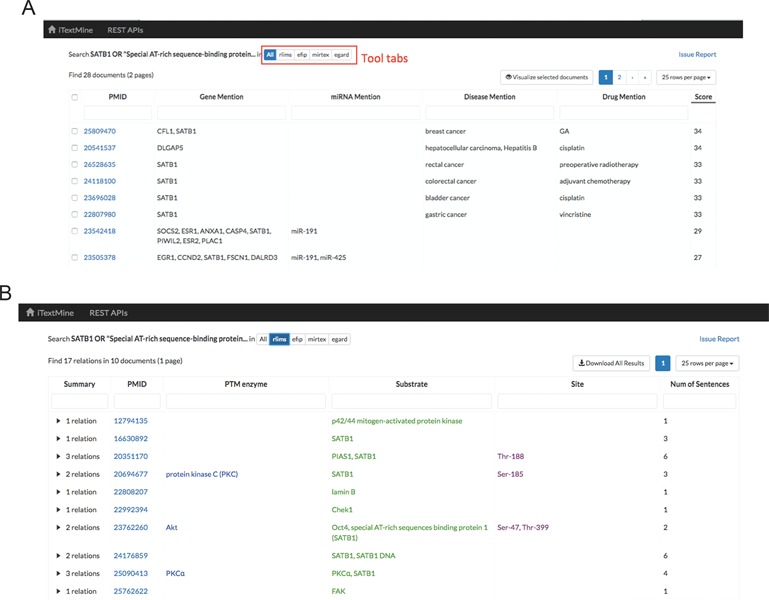
Tabular view of text-mining results. **(A)** Summarized results for query: SATB1 OR ‘Special AT-rich sequence-binding protein 1’. **(B)** Search result for RLIMS-P.

**Figure 4:**
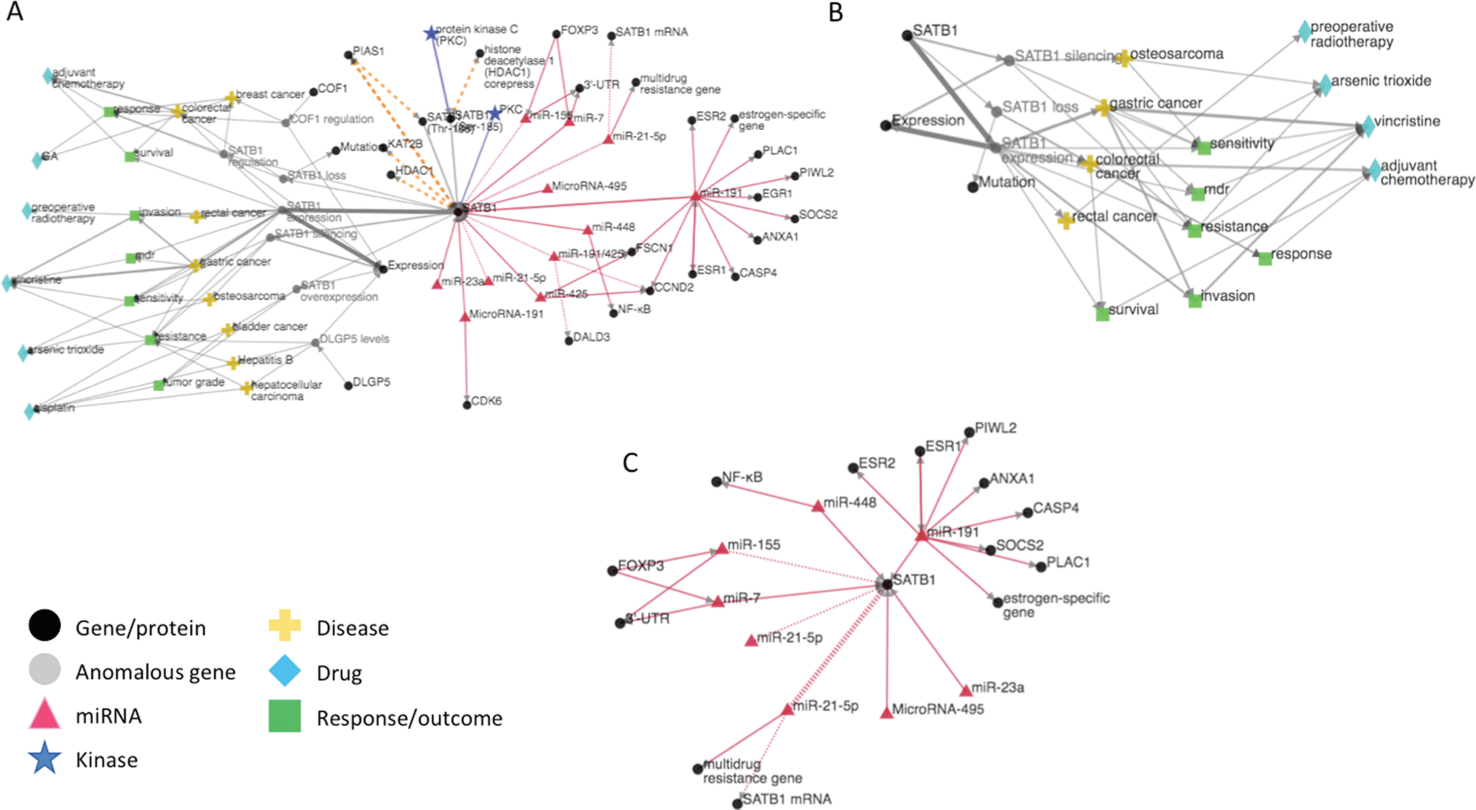
**(A)** iTextMine network for human SATB1. **(B)** Sub-network of the human SATB1 network focusing on therapeutic response. **(C)** Sub-network highlighting the SATB1 regulation by miRNAs.

**Figure 5:**
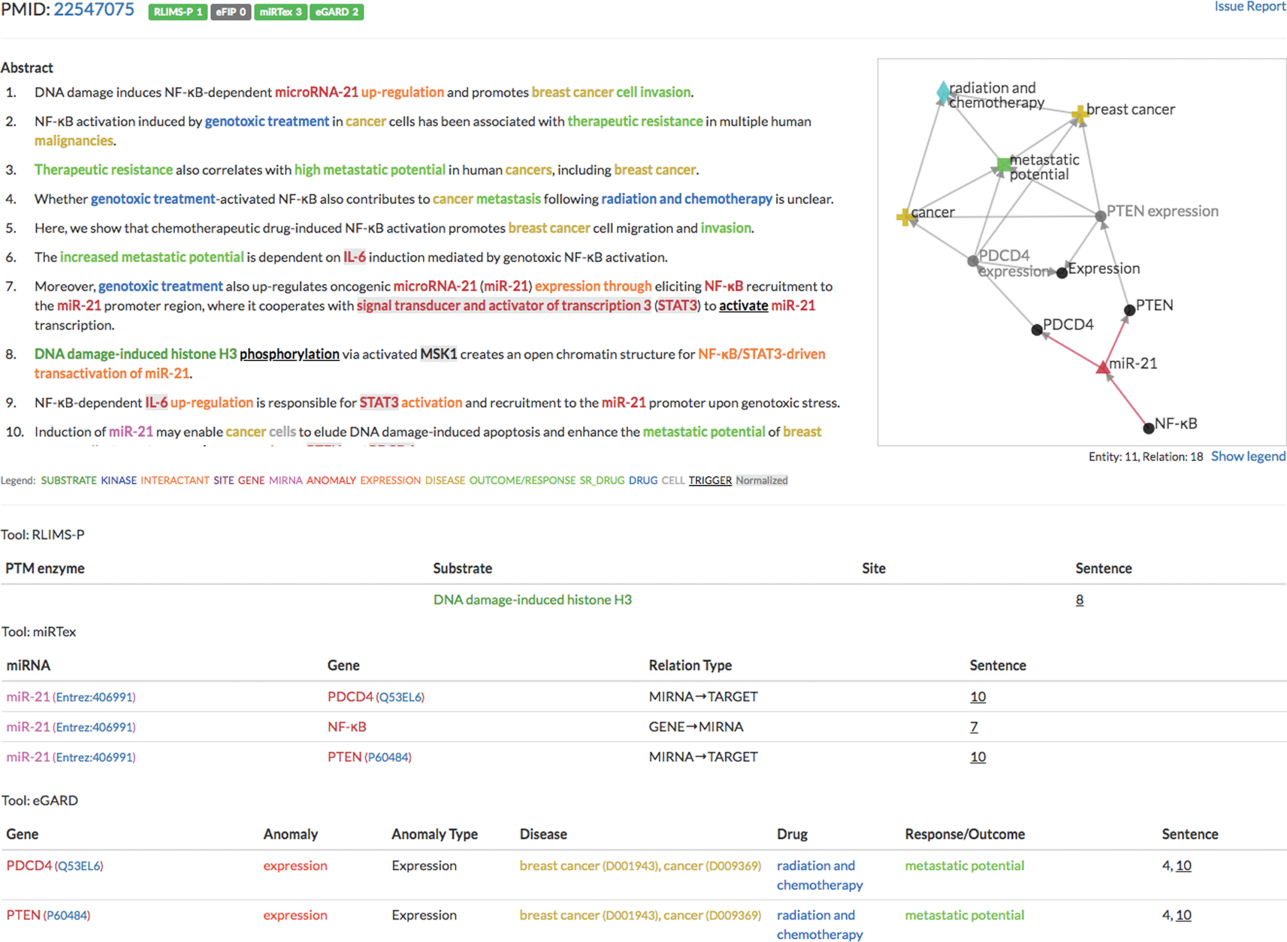
Example of the integration of multiple text-mining tools (PMID: 22547075).

#### Result presentations

Search results can be presented in two ways. First, if a user clicks the ‘Search’ button, a tabular view is generated. The summarized tab (Figure 3A) enumerates normalized gene mentions, normalized miRNA mentions, normalized disease mentions and drug mentions extracted by all of the tools. The PMIDs are ranked by the number of different types of entities extracted (those with most entity types extracted go first) and the sum of total relation counts. By checking the checkbox ahead of each document, the user can visualize the results extracted from these documents in a network. The tool tabs present relations extracted by each relation extraction tool. Figure 3B shows search results for RLIMS-P. Each row summarizes relations extracted from one abstract or article. The columns show the total number of relations extracted from the document, a link to the document view and the combined arguments from all the extracted relations. Each column is sortable and filterable. By clicking the right triangle at the beginning of each row, a list of individual relations is shown for further investigation.

The second way of browsing search results is through network visualization by clicking the ‘Visualize’ button. The extracted entities and relations are represented with nodes and edges in the network (Figure 4A). Entities are merged if they are normalized to the same ID. If normalized ID is missing, entities are merged by the same text mention. Relations are weighted by the amount of evidence; thicker edges represent relations extracted from more sentences or by more tools. Clicking each relation will trigger the left sidebar to display all text evidence. Users can explore the network by dragging nodes around and zooming in/out.

#### Document view

The document view shows the detailed text evidence of extracted entities and relations in one abstract or article (as Figure 5 in use case 1). The page has three sections: text evidence, network visualization and relations. The text evidence section displays the sentences with color-coded entities. The network view represents all the entities and relations with nodes and edges. The relation table lists relation arguments and attributes. The three sections are linked together; clicking a relation in the table will highlight the evidence sentence in the text section and highlight the corresponding edge in the network section.

#### Issue reporting

We set up a Google form for users to report issues. The link can be found in both the search and document pages. The report form asks users to provide their email, the PMID/PMCID, the text-mining tool name and describe the issue. The engineering team will be notified when an issue is submitted. They can review and assign it to corresponding developers.

#### Use case 1

We demonstrate the utility of iTextMine’s integration through a sample query for ‘PTEN AND breast cancer’ that finds 592 abstracts in the database with results from one or more of the four tools. Abstracts annotated by multiple tools provide enriched content. For example, PMID: 22547075 (27), as shown in Figure 5, is a cell line study on mechanisms of resistance to radiation and chemotherapy (genotoxic agents). Though the mechanisms for the interactions described in the paper are quite complicated, our system extracted multiple relations that capture the main aspects of the discussed model: (i) RLIMS-P captured that DNA damage-induced histone phosphorylation initiated the process leading to the NF-κB activation of microRNA miR-21; (ii) miRTex extracted the relationships in which NF-κB activates miR-21, which, in turn, represses expression of the PTEN and PDCD4 genes; (iii) eGARD extracted a multi-sentence relationship indicating that PTEN expression can regulate the metastatic potential of radiation and chemotherapy in breast cancer. Note the graphical display with the various relationships. This use case presents the general structure of relationships provided by the different tools in iTextMine. We acknowledge that not all relations in this complex mechanism were captured by the existing tools. For example, the potential role of the gene STAT3 in miR-21 activation was missed, as was the potential role of gene interleukin IL-6. This is inevitable because the tools we have currently implemented were designed for specific purposes, not the extraction of all possible relationships. However, having the results of multiple tools in one integrated system has captured much of the network, making needed improvements to existing tools or the need for new tools more apparent.

#### Use case 2

The DNA-binding protein SATB1 (also known as Special AT-rich sequence-binding protein 1) is a nuclear factor that functions as a global chromatin organizer to regulate gene expression [PMID: 16630892 (28), PMID: 22807980 (29)]. As a critical global regulator, its deregulation is found in many cancers. Since SATB1 has been implicated in drug resistance in many cancers [PMID: 22807980 (29), PMID: 24696710 (30), PMID: 19860849 (31)], we are interested in learning how SATB1 is regulated at the expression (miRNA) and post-translational modification (phosphorylation) levels and also about any anomalies of SATB1 (either expression or mutation) that have some impact on drug response to cancer. We hypothesize that controlling regulators of expression of SATB1 could be a potential point of intervention to overcome drug resistance.

Figure 4A shows the iTextMine network for human SATB1 based on the query ‘SATB1 OR “Special AT-rich sequence-binding protein 1”’. (The query also retrieves several results for mouse Satb1, which form a distinct network and are not shown.) The left side of the network illustrates the connection of SATB1 to drugs, disease and disease outcomes. The right side of the network indicates that SATB1 is highly regulated by miRNAs and phosphorylation events. Inspection of the outcomes (green nodes) reveals that several of them [e.g. response, resistance, multi-drug resistance (mdr)] relate to response to therapy. The sub-network based on the documents that contain these therapeutic response terms is shown in Figure 4B. From this network and the accompanying text evidence, we can see that SATB1 expression is a key factor in therapeutic response. For example, high expression of SATB1 contributes to vincristine resistance in gastric cancer [PMID: 22807980 (29)] and to arsenic trioxide resistance in osteosarcoma [PMID: 25317073 (32)]. Interestingly, loss of SATB1 expression is associated with a poor response to adjuvant chemotherapy in colorectal cancer [PMID: 24118100 (33)], indicating that the direction of the effect of SATB1 expression on drug response may be cancer type and/or therapy specific.

Given the central role of SATB1 expression levels in therapeutic response, it is very relevant that SATB1 is regulated by at least seven different miRNAs (Figure 4C). miRNAs affect gene expression, usually decreasing expression of their targets by preventing their translation. Thus, manipulating the levels of SATB1-specific miRNAs could potentially modulate the response to therapy. For example, increasing the level of one or more SATB1-specific miRNAs in osteosarcoma might improve the response to vincristine. Conversely, knocking down miRNA levels, so as to promote increased SATB1 expression, might be beneficial in colorectal cancer chemotherapy.

Finally, the phosphorylation information in the network provides insight into the mechanism by which SATB1 might affect drug response. Phosphorylation of SATB1 increases its interaction with histone deacetylases [PMID: 24176859 (34), PMID: 16630892 (28), PMID: 20694677 (35)] and decreases its interaction with the histone acetylase PCAF [KAT2B; PMID: 16630892 (28)]. These binding partners are involved in chromatin remodeling and affect the global gene expression pattern. Note that the combined RLIMS-P and eFIP outputs in iTextMine bring together the impact of phosphorylation on the PPI, the phosphorylation sites and regulating kinases when the information is available.

By integrating information of different types from multiple articles across the literature, iTextMine enables quick review of SATB1 function, the role of SATB1 in disease and upstream regulation of SATB1 expression, allowing the user to infer or suggest new modes of intervention to improve disease outcomes.

## Future work

We are working on incorporating more text-mining tools into the iTextMine system to provide more entity types and relations. Currently, PubTator is the only resource providing entity normalization information, but we are evaluating additional entity recognition and normalization tools that could be added to this system. For an entity that is identified and normalized by multiple NER tools, we may build a ranking mechanism to select the most confident result. Data validation using duplicate results generated by multiple tools is also an interesting option. The search interface will be improved to allow more flexible queries, such as searching entities by ID and performing relation-centric searches.

## Conclusion

In this paper, we describe the iTextMine system with an automated workflow to run multiple text-mining tools on large-scale text for knowledge extraction. We addressed challenges in system development by using parallel processing with dockerized text-mining tools, a common JSON output format and text alignment. iTextMine has been used to process all Medline abstracts and PMC open access full-length articles with four relation extraction tools. The website (http://research.bioinformatics.udel.edu/itextmine) allows users to browse the text evidence and view integrated results for knowledge discovery through network visualization.

## Notes

**Database URL:** http://research.bioinformatics.udel.edu/itextmine
